# Food Consumption Pattern and Dietary Diversity Among Pregnant and Lactating Women, Children, and Adolescent Girls in Devbhumi Dwarka District, Gujarat: A Cross-Sectional Study

**DOI:** 10.7759/cureus.28168

**Published:** 2022-08-19

**Authors:** Tanveer M Umallawala, Priyanka Shah, Tapasvi Puwar, Somen Saha, Apurvakumar Pandya, Mayur B Wanjari, Deepak Saxena

**Affiliations:** 1 Epidemiology and Public Health, Indian Institute of Public Health, Gandhinagar, IND; 2 Public Health and Community Medicine, Indian Institute of Public Health, Gandhinagar, IND; 3 Public Health, Indian Institute of Public Health, Gandhinagar, IND; 4 Public Health, Jawaharlal Nehru Medical College, Datta Meghe Institute of Medical Sciences (Deemed to be University), Wardha, IND; 5 Parul Institute of Public Health, Parul University, Vadodara, IND; 6 Research, Jawaharlal Nehru Medical College, Datta Meghe Institute of Medical Sciences (Deemed to be University), Wardha, IND

**Keywords:** gujarat, devbhumi dwarka, food frequency, dietary diversity, food consumption

## Abstract

Background

Food consumption patterns and dietary diversity are vital sources for the nutrition status of pregnant women (PW) and lactating women (LW), children, and adolescent girls. Undernutrition, food consumption pattern, and poor dietary diversity are interlinked; however, not much is known in the context of rural Gujarat. This study aims to assess the regional pattern of food consumption and dietary diversity among pregnant and lactating women, children, and adolescent girls from Devbhumi Dwarka District in the state of Gujarat.

Methods

A cross-sectional study was conducted in four blocks of Devbhumi Dwarka District of Gujarat. A cluster sampling method was used for a better representation. A total of 632 pregnant women, 562 lactating mothers, 855 children aged 7-24 months, and 1,252 adolescent girls were assessed for food consumption patterns.

Results

Consumption of cereals (98%) was found to be highest among pregnant women, whereas consumption of pulses and fruits, which are rich in proteins and vitamins, was inadequate. Overall, the consumption of fruits was inadequate among adolescent girls (56.5%). Moreover, inadequate consumption of green leafy vegetables (36.4%) was noted among children. The dietary diversity score (DDS) for the study population ranges between 4.5 and 4.8, indicating medium diversity in food.

Conclusion

Cereal consumption is higher, which indicates a major part of the energy consumed by vulnerable groups. In contrast, low consumption of pulses, fruits, milk, and green leafy vegetables suggests the possibility of one or more micronutrient deficiencies. There is a need for innovative intervention to change food habits and promote locally available nutrient-rich food and awareness of the importance of various food groups to improve food patterns and the health of vulnerable groups.

## Introduction

An adequate food consumption pattern and dietary diversity are critical for the overall growth and development and health outcomes of pregnant women (PW) and lactating women (LW), children, and adolescent girls [1]. The recent Global Nutrition Report 2020 revealed that every third child in India is malnourished and expressed concerns regarding achieving the Global Nutrition Targets 2025 [2].

As per the National Family Health Survey 4 data, 38.4% of children under five years of age are stunted, 21% of children are wasted, and 35.8% are underweight in India [3]. The statistics of Gujarat are more concerning; about 38.5% of children under five years of age are stunted, 26.4% are wasted, and 39.3% are underweight [3].

Issues of malnutrition cannot be achieved without understanding and focusing on food consumption patterns and dietary diversity among children, adolescent girls, pregnant women, and lactating mothers [4,5]. Many organizations advocate for dietary diversity strategies to tackle the burden of micronutrient malnutrition among said vulnerable groups [6].

Most of the previous research on malnutrition worldwide, particularly in India, focused on anthropometric failures rather than dietary diversity [7,8]. The dietary diversity and food consumption pattern in households is an indirect measure of diet quality and meeting of the nutritional needs of households [9]. Dietary diversity indicates a person’s access to various food sources and thus reflects the adequacy of nutrients, household and individual food security, nutrient intake, and agricultural biodiversity of a particular region [10].

Regional socioeconomic status and cultural diversity impact the diet diversities of the population. People in Gujarat have predominantly a vegetarian diet where milk and fruits are consumed universally and a considerable population eats meat and eggs. Consumption of milk and fruits varies across regions and socioeconomic classes. Previous studies highlight that insufficient nutrient intake, poor dietary diversity before and during pregnancy, and the lactation period can affect both women and children [7-10].

Studies on food consumption and dietary diversity are limited in the context of Gujarat state. As per the baseline assessment of Project Tushti in Devbhumi Dwarka [11], the majority of occupations in the district are agriculture, animal husbandry, fishing, and petty trading. The staple diet of the population has been bajra rotla (millet bread), rice, dal, and potato. It was noted that most people, including women, chew tobacco and snuff, which is locally called “faki.” Daily wagers’ food consumptions were dependent on daily wages and reportedly had limited access to procure nutritious food, which may result in insufficient food consumption and dietary diversity. The baseline assessment also revealed a lack of knowledge about malnutrition, dietary diversity, and food groups. Moreover, considering the new district (separated from Jamnagar District on August 15, 2013), studies in the public health and nutrition domains are negligible. In view of this, the present study was conducted to assess food consumption patterns and dietary diversity among pregnant and lactating women, adolescent girls, and children of Devbhumi Dwarka District in Gujarat state.

## Materials and methods

Study setting and design

In this study, a descriptive cross-sectional design was used. The study was conducted in four blocks, namely, Khambhaliya, Bhanvad, Kalyanpur, and Dwarka, in the Devbhumi Dwarka District of Gujarat.

Study population

The sample size for the study was 3,292, which included 623 pregnant women and 562 lactating women, 855 children (7-24 months of age), and 1,252 adolescent girls.

Data collection

For the assessment of food consumption patterns, children aged 0-6 months were excluded from the study, as there is no food consumption at that stage. The sample for pregnant and lactating women was separated from the study because of the changes in the food consumption pattern. A common tool for 24-hour dietary recall was used for all beneficiaries, and key informant interviews were conducted with mothers of children aged 7-24 months, pregnant and lactating women, and adolescent girls. The 24-hour dietary recall is a dietary assessment tool consisting of a structured interview. The tool intends to capture detailed information about all foods and beverages (type, frequency, and groups of foods) consumed by the study subjects in the past 24 hours. The food groups categorized were cereals, pulses, green leafy vegetables, fruits, milk and milk products, groundnut and oilseeds, and non-vegetarian items, including eggs [12]. The food items in cereals included wheat, bajra (pearl millet), jowar (sorghum), rice, and rice flakes. Pulses included arhar dal (pigeon pea), urad dal (black lentils and black gram), masoor dal (red lentils), and legumes. Green leafy vegetables included fenugreek leaves, spinach, and vegetables that are green in color such as cluster beans and cabbage. Fruits included all fruits that are available locally such as banana, papaya, pineapple, orange, apple, and sapodilla (locally known as chikoo). Milk and milk products include milk, buttermilk, curd, and cheese. Groundnut and oilseeds include oils such as coconut oil, groundnut oil, and cottonseed oil. Non-vegetarian items included meat, chicken, eggs, and fish.

Dietary diversity measures food security and helps monitor changes and impacts, particularly when resources available for such measurement are scarce [4]. Low dietary diversity may suggest chronic nutrient deficiencies. In the present study, the women’s dietary diversity score (WDDS) was calculated based on 24-hour recall and the number of food groups consumed and reflects the probability of micronutrient adequacy of the diet using FAO 2011 guidelines [13]. Study subjects were interviewed using a survey questionnaire consisting of subjects’ basic information, nine food groups, and 24-hour recall tool. After data collection, all the food groups consumed by each subject were added upon, and the score was calculated. In this study, WDDS was calculated for 1,238 adolescent girls and 1,180 pregnant (n=620) and lactating (n=560) women. The dietary diversity score for children could not be calculated because the required food groups could not be extracted from the data. According to the guidelines, a dietary diversity score of less than 3 indicates low dietary diversity, 4-5 indicates medium, and greater than equal to 6 indicates high dietary diversity [13].

Ethical consideration

The study subjects sought written consent, and formal approval was obtained from state and district authorities. The Institutional Ethics Committee of the Indian Institute of Public Health, Gandhinagar, approved the study (IEC/IRB approval number 14/2019-20).

## Results

The total sample for assessing the food consumption pattern is 3,292, including 623 pregnant women and 562 lactating women, 855 children, and 1,252 adolescent girls. Across four blocks of the district, the majority of the sample was covered from Dwarka Block.

Consumption of food groups among pregnant women (PW)

Consumption of cereals (98%) was the highest among pregnant women. Table 1 shows the consumption of food groups among pregnant women within the past 24 hours of the survey. The cereals commonly consumed were bajra, jowar, wheat, rice, semolina, and rice flakes. The common preparations from these cereals were roti, rotla, bhakhri, poha, sheera, khichdi, and bhaat. Along with cereals, other food groups that were consumed more were groundnut and oilseeds (89.6%), followed by milk and milk products (82.6%), green leafy vegetables (71%), pulses (61.6%), and fruits (59%).

**Table 1:** Consumption of Food Groups Among PW (N=623)

Food groups	Total
Cereals	617 (97.6%)
Pulses	389 (61.6%)
Green leafy vegetables	449 (71%)
Fruits	373 (59%)
Milk and milk products	522 (82.6%)
Groundnut and oilseeds	566 (89.6%)
Non-vegetarian items including eggs	43 (6.8%)

There was 100% consumption of cereals in all blocks except Dwarka (96.8%). Conversely, consumption of pulses (61.6%) and fruits (59%) were restricted. Red gram, dhal-tuver dal, green gram, and dhal-mug dhal were noted as part of pulses, whereas apple, banana, and grapes were consumed as part of fruits. The majority (82.6%) of the subjects consumed buttermilk, milk, and curd as part of milk and milk products.

Notably, 71% of the subjects consume green leafy vegetables. Comparing the consumption of fruits across four blocks, 65.2% of consumption was recorded in Dwarka, followed by Kalyanpur with 65% and Bhanvad with 57.3%. In contrast, only 50.3% of the subjects consumed fruits in the Khambhaliya Block, making them the least consumed. Animal origin foods, including fish, prawns, and chicken, along with eggs, were consumed less overall by 6.8% of the subjects; the majority of consumption (18.1%) was seen in the Dwarka Block.

Consumption of food groups among lactating women (LW)

Consumption of food groups among lactating women is shown in Table 2, which indicates a similar scenario for pregnant women. The majority of the LW consumed cereals (98%) and groundnut and oilseeds (90.9%). Less than 50% of LW consumed fruits. It was seen that milk consumption was higher in LW (82%) in the form of milk, buttermilk, curd, and tea. Animal origin foods such as fish, chicken, and eggs were consumed more in the Dwarka Block by LW (6%). There is a lack of fruits and slightly low consumption of green leafy vegetables and pulses in the diet.

**Table 2:** Consumption of Food Groups Among LW (N=562)

Food groups	Total
Cereals	552 (98.2%)
Pulses	395 (70.3%)
Green leafy vegetables	417 (74.2%)
Fruits	269 (47.9%)
Milk and milk products	458 (81.5%)
Groundnut and oilseeds	511 (90.9%)
Non-vegetarian items including eggs	37 (6.6%)

The mean women’s dietary diversity score (WDDS) of Devbhumi Dwarka for PW and LW was 4.8. Out of 1,185 PW and LW, the DDS for only 1,180 women could be calculated. Table 3 presents the dietary diversity scores of PW and LW.

**Table 3:** Dietary Diversity Score of Pregnant Women and Lactating Women

Dietary diversity	Pregnant women (n=620)	Lactating women (n=560)	Total (N=1,180)
Low dietary diversity	78 (12.6%)	86 (15.4%)	164 (13.9%)
Medium dietary diversity	335 (54%)	302 (53.9%)	637 (54%)
High dietary diversity	207 (33.4%)	172 (30.7%)	379 (32.1%)

The results of WDDS showed that the majority (54%) of the women in Devbhumi Dwarka had medium dietary diversity, followed by 32.1% of women having high dietary diversity and 13.9% having low dietary diversity. The diversity of pregnant and lactating mothers shows almost no difference compared to their increasing biological needs.

Consumption of food groups among children aged 7-24 months

As shown in Table 4, in most cases (89.7%), cereals such as wheat flour, rice, and items such as roti, khichdi, paratha, rotla, and biscuits were primarily given to children. Cereal consumption in the Dwarka Block is high as compared to other blocks.

**Table 4:** Consumption of Food Groups Among Children Aged 7-24 Months (N=855)

Food groups	Total
Cereals	767 (89.7%)
Pulses	510 (59.6%)
Green leafy vegetables	311 (36.4%)
Fruits	475 (55.6%)
Milk and milk products	671 (78.5%)
Groundnut and oilseeds	588 (68.8%)
Non-vegetarian items including eggs	77 (9%)

Animal origin food items, including fish, mutton, chicken, and eggs, were consumed overall by 9% of the respondents. The majority of consumption (16.2%) was seen in the Dwarka Block. Overall, inadequate consumption of green leafy vegetables (36.4%) was noted among children. They primarily consume only fenugreek leaves, spinach, etc. Among the four blocks, the lowest consumption (26.2%) was observed in the Khambhaliya Block compared to the Dwarka Block (34.7%), Bhanvad Block (42.8%), and Kalyanpur Block (40.2%). Green leafy vegetables are a good source of iron and other vitamins, but poor consumption may prevent children from benefiting from their overall growth and development. Nearly half of the children (57.6%) consume pulses and fruits. In pulses, green gram, Bengal gram, red gram dhal, green gram dhal, etc. were commonly consumed, whereas apple, banana, grapes, and orange were the typically preferred fruits. Overall, the Khambhaliya Block is the only block with low consumption of cereals, pulses, green leafy vegetables, and milk/milk products among children, which can be considered for appropriate intervention.

Consumption of food groups among adolescent girls

As presented in Table 5, 99.3% of cereal consumption among adolescent girls was observed. Wheat flour, bajra flour, rice, etc. were routinely used in their diet. These were used to prepare roti, rotla, bhakhri, khichdi, bhaat, puri, thepla, etc. About 78% of consumption of milk and milk products and groundnut and oilseeds were observed among the girls. Milk and buttermilk were the most common milk and milk products consumed by the girls.

**Table 5:** Consumption of Food Groups Among Adolescent Girls in the Last 24 Hours (N=1,252)

Food groups	Total
Cereals	1,243 (99.3%)
Pulses	807 (64.5%)
Green leafy vegetables	853 (68.1%)
Fruits	708 (56.5%)
Milk and milk products	978 (78.1%)
Groundnut and oilseeds	928 (74.1%)
Non-vegetarian foods	153 (12.2%)

In Kalyanpur, most girls (86.9%) were consuming milk and milk products compared to other blocks. This is a good practice as milk and milk products are rich in calcium, which helps in bone development. Mostly, the girls use cottonseed oil, groundnut oil, corn oil, etc. for cooking food. More than half of the adolescent girls (66.3%) consumed pulses and green leafy vegetables. Green gram dhal, Bengal gram dhal, peas, red gram dhal, etc. were the commonly consumed pulses, while green leafy vegetables such as fenugreek leaves, shepu, and spinach were primarily eaten. Nearly one-third of the girls (78.67%) in the Bhanvad Block consumed pulses and green leafy vegetables compared to other blocks.

Overall, the consumption of fruits was inadequate among adolescent girls (56.5%). The highest fruit consumption was observed in the Bhanvad Block among adolescent girls (68.3%) compared to other blocks. In Dwarka, nearly a quarter of the adolescent girls (22.4%) consume non-vegetarian items such as fish, egg, chicken, and meat, which is the highest compared to other blocks. In summary, the majority of adolescent girls from the Bhanvad Block consume pulses, green leafy vegetables, and fruits, whereas the highest number of milk/milk products were consumed in Kalyanpur.

The dietary diversity of 1,238 adolescent girls could be calculated from the total sample of 1,252. Table 6 presents the dietary diversity score. Their mean WDDS came out to be 4.5, indicating medium dietary diversity. In the present study, an almost equal percentage of girls having low dietary diversity (23.7%) and high dietary diversity (27.3%) was observed.

**Table 6:** Dietary Diversity Score of Adolescent Girls

Dietary diversity	Adolescent girls (N=1,238)
Low dietary diversity	293 (23.7%)
Medium dietary diversity	607 (49%)
High dietary diversity	338 (27.3%)

## Discussion

The consumption of food patterns in Devbhumi Dwarka was assessed for all vulnerable groups such as children, adolescent girls, PW, and LW. The results showed high consumption of cereals across all groups and very low consumption of animal origin food items [14]. Consumption of green leafy vegetables and fruits was also considerably lesser. A previous study conducted by Gupta et al. concluded that in rural India, meat consumption is relatively low, and higher consumption of animal-origin food is mainly seen in the western coastal region [15]. The Devbhumi Dwarka District has a coastal region; however, the consumption of animal origin food items (such as meat, chicken, fish, and eggs) was considerably low (6.8%).

The study results show that most subjects relied mostly on starchy-based diets, with 93% of them consuming this food group. The high consumption of starchy diets could be explained by the high production of starchy-based cereal crops such as pearl millet, sorghum, and maize that are often used to prepare staple traditional dishes such as roti, rotla, bhakhri, poha, sheera, khichdi, and bhaat. Consumption of pulses and fruits (which are rich in proteins and vitamins) was below FOA recommendations [13]. There was a low consumption of green leafy vegetables and pulses in the diet of LW. About 71% of subjects (pregnant and lactating women and adolescent girls) reported consumption of green leafy vegetables. However, green leafy vegetable consumption was very low among children; only 36.4% of the children reported consuming green leafy vegetables. Consumption of fruits among adolescent girls was also limited; about 43.5% of the adolescent girls reported not consuming fruits. Similar findings were reported in previous studies [15,16]. The study showed that the intake of pulses and green leafy vegetables was below the recommended among the rural population [15].

Geographic variation in food consumption is very well documented in many Indian and global literature [16-19]. Wide variation in food habits was noted across blocks. PW from all blocks had the highest consumption of cereals, except Dwarka. The Dwarka Block had the highest consumption of animal origin foods such as fish, chicken, and meat, including eggs. Bhanvad had the highest milk consumption. Consumption of pulses and fruits was reported to be low across blocks among both PW and LW, although highly recommended during pregnancy and lactation. Dietary diversity provides adequate energy, macronutrient, and micronutrient to support fertility, pregnancy, positive birth outcomes, and the future health of the children [13,20]. In the present study, the mean women’s dietary diversity score (WDDS) of Devbhumi Dwarka for PW and LW was 4.8, indicating moderate dietary diversity.

Both moderate and medium dietary diversity reflects a deficiency of macronutrients and micronutrients. The adolescence period, pregnancy, and lactation are the stages that require more nutrients. Also, studies have shown that pregnant women with moderate and low dietary diversity have a significantly higher proportion of preterm birth, low birth weight, and stillbirth [16,20]. Many studies on dietary diversity have reported negative consequences of poor dietary diversity on overall health, immunity, mental health, and reproductive and social capabilities [18-22]. Serious effects such as night blindness and cognitive impairment, mostly among children under five years and pregnant women, were also documented [1,2,20].

The present study provides insight on possible targeted interventions for changing food habits, identifying local nutritious food, and awareness on growing nutritious fruits and vegetables, poultry, and livestock to improve the food diversity of vulnerable groups in the district. Interventions should incorporate an understanding of the socioeconomic setting, occupations, food market of the target area, and its population.

Moderate and low dietary diversity across pregnant and lactating women, children, and adolescent girls in the district can be addressed by designing and implementing behavior change interventions to modify food habits in the community through promoting locally available nutritious food and generating awareness of the importance of various food groups among different age groups. Moreover, awareness regarding the use of many government programs and supplies such as ration and take-home ration packets from Anganwadi centers along with dietary counseling to pregnant and lactating women and adolescent girls can be promoted to reduce the chances of deficiency and improve nutritional practices.

Limitation of the study

Dietary diversity for children could not be calculated due to the lack of categorization into 11 food groups required to calculate the score. However, the consumption pattern of different food groups was looked upon. The food groups that identified the consumption pattern were cereals, pulses, green leafy vegetables, fruits, milk and milk products, nuts and oilseeds, and non-vegetarian items and poultry. The 24-hour dietary recall was taken for one day only. A three-day 24-hour dietary recall has been argued as more accurate to depict consumption patterns. Despite these shortcomings, the current study contributes to a limited but growing literature on food consumption patterns and dietary diversity among vulnerable groups in the district. It provides programmatic insights for improving nutritional practices in the district.

## Conclusions

Consumption of less diversified food among pregnant and lactating women, children, and adolescent girls is common in the study area. Cereal consumption is higher, which indicates a significant part of the energy consumed by vulnerable groups. Still, consumption of pulses, fruits, milk, and green leafy vegetables was low, indicating the possibility of one or more micronutrient deficiencies. Focusing on intervention to change food habits through locally available nutritious food, awareness of the importance of various food groups among different age groups can be beneficial. The findings from this study have not been published or presented in any other manuscripts submitted.
